# Psychedelic Art and Implications for Mental Health: Randomized Pilot Study

**DOI:** 10.2196/66430

**Published:** 2024-12-03

**Authors:** Mary L Peng, Joan Monin, Polina Ovchinnikova, Amanda Levi, Terika McCall

**Affiliations:** 1 Social and Behavioral Sciences Department Yale School of Public Health New Haven, CT United States; 2 Department of Global Health and Social Medicine Harvard Medical School Boston, MA United States; 3 Department of Orthopedics and Sports Medicine Boston Children's Hospital Boston, MA United States; 4 Division of Health Informatics, Department of Biostatistics Yale School of Public Health New Haven, CT United States; 5 Department of Biomedical Informatics and Data Science Yale School of Medicine New Haven, CT United States; 6 Center for Interdisciplinary Research on AIDS (CIRA) Yale School of Public Health New Haven, CT United States

**Keywords:** digital art, mental health, psychedelic art, well-being, pilot trial, digital health tool, art therapy

## Abstract

**Background:**

Psychedelic art (PA) emerged in the 1960s during the psychedelic era; then characterized by visuals induced by the ingestion of psychedelic drugs, it is now an art form known for its vibrant colors, distorted forms, and intricate patterns. Building upon the existing research on art viewing as an effective means to improving physiological and psychological well-being, viewing PA is postulated to evoke positive emotions and provide a meditative experience, contributing to improved mental well-being.

**Objective:**

This study aims to investigate how digitally rendered PA influences viewers’ perceived emotional, mental, and physical states compared to imagery of natural scenery, offering insights into potential applications in mental health care and well-being.

**Methods:**

Overall, 102 participants age 18 to 35 years were randomly assigned to either the experimental group viewing 300 seconds of PA imagery (50/102, 49%) or the control group viewing 300 seconds of scenic imagery (52/102, 51%), after which every participant completed a survey that gathered qualitative data on the perceived impact of viewing their given imagery on their physical, mental, and emotional states through open-ended questions. Thematic analysis was conducted to identify the patterns of experiences reported by the participants.

**Results:**

Qualitative analysis unveiled a greater intensity and diversity of emotional, mental, and physical impacts induced by PA compared to natural scenery, including the sense of relaxation and peace, anxiety and stress alleviation, joy, thrill and sense of euphoria, sensations of awe and wonder, hypnotizing effect, holistic meditative effect, provocation of creative thoughts, induced hyperawareness of bodily states, and transitions from induced overstimulation or anxious thoughts to feelings of calmness.

**Conclusions:**

The preliminary findings of this study suggest that PA is a rich and complex form of visual art that has the potential to facilitate healing and promote well-being and mental health. PA presents promising avenues for integration into mental health care, therapeutic practices, digital health, health care environment, and medical research.

## Introduction

### Background

Psychedelic art (PA) conventionally refers to artwork manifested in the context of ingesting lysergic acid diethylamide (LSD)–type drugs and related substances [1]. Psychedelic broadly means “mind manifesting” or “mind revealing” [2]. In the parlance of contemporary art, a more expansive definition of PA includes any art, graphics, and visual displays that aim to depict mind manifestation and the inner world of the human psyche without necessarily involving any substance use. Characteristics of PA include the use of kaleidoscopically swirling color patterns, highly distorted or surreal visuals, bright colors, and motions of abstract shapes to evoke and enhance psychedelic states of consciousness [3].

There is a wealth of research on the interconnection between health and art, including music and visual art, delineating the impact of artistic engagement as effective means to alleviating physiological and psychological symptoms [4]. Improved health outcomes, such as improved well-being and reduced negative emotions [5], reduced depression and hemodialysis parameters [6], reduced stress and anxiety [6], improved apical heart rates [7], and improved sense of well-being and relaxation and reductions in tension and serum cortisol levels [8], have all been associated with art-based interventions. Study populations of art-based interventions span individuals with a wide array of conditions, such as coronary artery disease [9], compassion fatigue [10], chronic illnesses and disability [11], and HIV [12].

The exploration of the effects of viewing art on mental health has gained traction in recent years. Research has shown that the mere presence of art in one’s surrounding or the simple act of observing art can yield benefits for mental health [13], reducing stress and providing psychological benefits [14,15]. One randomized controlled trial revealed that patients with hematologic cancer who engaged in bedside art viewing experienced reduced feelings of anxiety, depression, and improved mental health states [16]. The esthetic experience of art can thus be considered as a form of psychological respite, fostering emotional well-being in clinical settings. Research has further suggested that esthetic experiences, associated with neural activation across multiple sensory modalities, extend beyond conceptions of conventional artworks and apply to all mechanisms of perceiving visual stimuli, positively affecting individuals’ sense of well-being through rewarding neural feedback [17]. While currently there is a lack of research on the impact of PA on mental health, the vibrant and fantastical visual components characteristic of PA can be expected to benefit individuals’ well-being through the shared mechanism of esthetic appraisal.

Despite ample evidence regarding the impact of art on mental health and other health outcomes and the widespread discussion on social media about people using PA videos to induce sleep, alleviate stress, achieve calmness, and increase mental clarity, there remains limited research on how viewing PA affects one’s physiological and mental states. This study thus sets out to address this gap by systematically measuring people’s feedback in response to the experience of viewing digitally rendered PA. To the best of the authors’ knowledge, this is the first study that explores this subject matter in a controlled setting.

### Objectives

With the advancement of digital art, art-based health interventions can now incorporate immersive multimodal features, such as animation and audio. Artistic features of PA can now be easily created without any substance use, and multimodal features can be added to visual displays to enhance the digital PA experience. Results from this study can thus not only offer valuable insight into the incorporation of digital PA as a wellness feature in consumer health products, such as meditation, mental health, and well-being applications, but also provide potential directions for innovative art therapies.

This study aimed to illuminate the currently unclear impact of PA. By conducting qualitative analysis using data from a randomized controlled trial, this study aims to empirically explore any distinct impact of PA on participants’ emotional, mental, and physical states compared to the control group.

## Methods

### Ethical Considerations

All data collected for this study were recorded by the principal investigator, MLP, in such a manner that the identity of the participants cannot readily be ascertained, directly or through identifiers linked to the participants. This study was approved by the Yale University Institutional Review Board (approval 2000033683). All participants signed consent forms including detailed declaration of study-related benefits and risks before participating in the study. No adverse events, defined as any occurrence of physical or mental distress symptoms among participants during the study that require clinical attention, occurred. The study was performed according to the ethical standards of human participant research set by the 1964 Helsinki Declaration. All participants received US $10 compensations in the form of Amazon gift cards upon completion of the study.

### General Design and Recruitment

The randomized pilot trial collected data from all participants regarding the perceived emotional, mental, and physical impacts of viewing their assigned imagery. The study was conducted in an enclosed, private room to ensure a controlled and distraction-free environment for each participant.

Recruitment for the study commenced on October 1, 2022, and data collection ended on October 26, 2022. Participants were recruited from a university in the Northeastern United States for this study. To be eligible for the study, an individual must be between the age of 18 and 35 years and able to communicate in English. Exclusion criteria included having severe visual impairment or blindness or having clinically diagnosed or self-identified cognitive impairment. The exclusion criteria were designed to minimize confounding effects that visual and cognitive impairments might have on the emotional, mental, and physical responses to visual stimuli [18]. Any participant who signed up, passed the eligibility screening, and consented was included in the study.

This study enrolled 102 participants. Out of the 110 who were recruited, 1 (0.9%) individual did not pass the eligibility screening. Due to conflicting schedules and incidents of illness, 5 (4.5%) individuals had dropped out before randomization was performed. Using a random number generator, the principal investigator (MLP) randomized 104 individuals to the experimental PA group (n=52, 50%) and the scenery control (SC) group (n=52, 50%). Two participants in the initial PA group dropped out of the study less than 12 hours before the experiment due to personal injury or illness, leaving the final experimental PA group with 50 participants.

### Data Collection

Research has shown that age [19,20], gender [21,22], stress level [23], sleep quality [24,25], and mental health conditions [26,27] could affect one’s visual and emotion processing capacity. Therefore, before the intervention, namely viewing the PA or scenery video, the participant completed a self-administered, web-based questionnaire to collect demographic information such as age, self-identified gender, self-identified race, recent stress level, and sleep duration and quality the night before the experiment to establish a comparable baseline among the participants. Specifically, age, gender, and mental health conditions were assessed based on participants’ self-reporting, indicating whether they had been diagnosed with any mental health conditions and specifying the diagnoses if applicable. A 5-point Likert scale was used to assess recent stress level, with 1 being “not stressed at all” and 5 being “very stressed,” and sleep quality, with 1 being “very bad” and 5 being “very good.”

**Figure 1:**
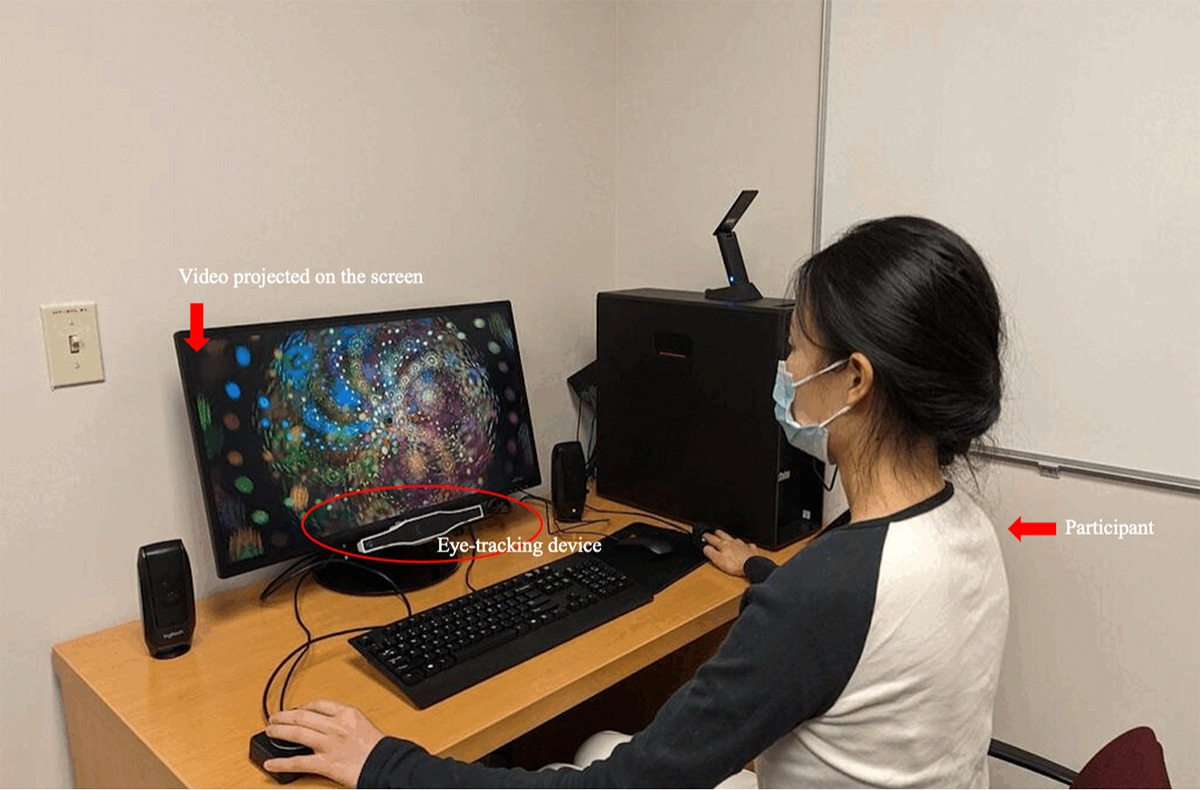
Experiment device setup of eye-tracking device, screen, and participant.

In the Consumer Health Informatics Lab where the study was conducted, temperature, lighting, and the distance between the participant and the screen were kept constant for all study sessions, whereby an eye-tracking device (Gazepoint version 3.0) was also used to ensure that all participants were focusing on the imageries throughout the experiment by calibrating and following their gaze paths throughout the experiment (refer to Figure 1 for demonstration of the setup). Each participant viewed a 300-second video of imagery according to their group allocation (PA and natural scenery). The PA imageries focused on vibrant colors and intricate patterns that evoke altered perceptions and consciousness. These elements were chosen based on existing research that suggests such visuals can stimulate emotional and cognitive responses [28]. The abstract pattern compositions and distortions were included to reflect visions akin to those reported by individuals experiencing hallucinogen-induced visions, without the need for drug intake [29]. In contrast, the natural scenery montage was designed with calm, familiar landscapes that have been shown in previous studies to promote relaxation [30]. The creative choices were ultimately to ensure the visual contrast between the 2 groups. The 2 videos can be accessed through the link provided in Multimedia Appendix 1. After watching the video, each participant completed a survey with open-ended questions to report the perceived impact of the imagery on their emotional, mental, and physical states. Specifically, they were asked (1) “please describe in as much detail as possible your physical, emotional, and mental states and the thoughts you had while watching the video;” and (2) “did you experience any negative sensations or emotions while watching the video. If yes, please describe in detail how you felt.”

### Data Analysis

To compare the demographic baselines of the control and experimental groups, descriptive statistics were calculated to summarize participants’ demographic information. This included information on age, gender, race, recent stress levels, sleep duration and quality the night before the experiment, and the presence of mental health conditions.

Thematic analysis was conducted to identify and analyze patterns within the open-ended questions using the attached interview guide. Three researchers (MLP, AL, and PO) independently coded the data and then compared their findings to identify common themes. The analysis followed the six-phase framework proposed by Clarke and Braun [31]: (1) familiarization with the data through repeated reading and noting down initial ideas; (2) generating initial codes by systematically sorting distinct codes of the data across the entire dataset; (3) searching for themes by collating codes into potential themes; (4) reviewing themes to ensure they accurately reflected the coded extracts; (5) defining and naming themes by ongoing analysis to refine the specifics of each theme and the overall story the analysis tells; and (6) producing the findings by selecting vivid, compelling extract examples, conducting a final analysis of selected extracts, and relating the analysis back to the research question and literature. Any discrepancies in coding were resolved through discussion until consensus was reached. This process involved multiple rounds of coding to refine and finalize the themes. Data saturation was considered achieved when no new themes or subthemes emerged from the data, indicating that the collected data adequately captured the participants’ experiences.

## Results

### Participant Characteristics

**Figure 2:**
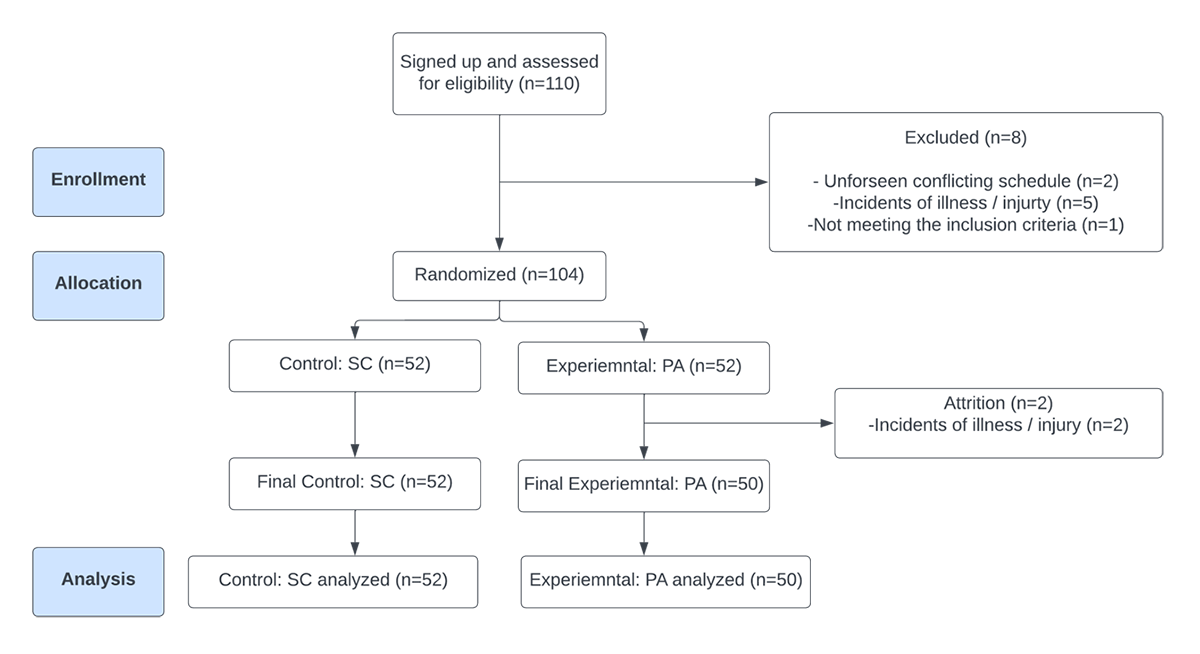
CONSORT (Consolidated Standards of Reporting Trials) diagram of enrollment, randomized allocation, and analysis. PA: psychedelic art; SC: scenery control.

The final study population included 102 young adults between the age of 18 and 35 years (PA group, n=50, 49%; and SC group, n=52, 51%; Figure 2). When estimating the sample size for a pilot trial, this study followed the general flat rule to use a minimum sample size of 12 participants per treatment arm [32]. The study well exceeded the recommended minimum sample size.

Participants’ characteristics, including age, self-identified gender, self-identified race, recent stress level, sleep duration and quality, and self-reported clinically diagnosed mental health conditions, are documented in Table 1. The PA group was nearly identical in age (mean 24.6, SD 3.1 y) as the SC group (mean 24.4, SD 2.6 y). The gender distribution across the PA group (39/50, 78% woman; 9/50, 18% man; 2/50, 4% nonbinary) and the SC group (38/52, 73% woman; 12/52, 23% man; 2/52, 4% nonbinary) was similar. The PA group (mean 3.5, SD 0.8) was comparable in recent stress level as the SC group (mean 3.3, SD 1.0). Sleep duration was also comparable between the PA group (mean 7.1, SD 1.0 h) and the SC group (mean 6.8, SD 1.1 h). Average sleep quality the night before the experiment was identical between the PA group (mean 3.5, SD 0.8) and the SC group (mean 3.5, SD 0.8). Within the PA group, 64% (32/50) reported 0 clinically diagnosed mental health condition, 18% (9/50) reported 1 condition, 12% (6/50) reported 2 conditions, and 6% (3/50) reported >3 conditions, and a similar distribution was observed in the SC group, whereby 65% (34/52) reported 0 condition, 17% (9/52) reported 1 condition, 10% (5/52) reported 2 conditions, and 8% (4/52) reported >3 conditions.

**Table 1:** Demographic baseline characteristics of study participants.

Characteristic			Total sample (N=102)		Experimental group: psychedelic art (n=50)		Control group: scenery (n=52)
**Age (y), mean (SD)**			24.5 (2.8)		24.6 (3.1)		24.4 (2.6)
**Gender, n (%)**							
	Woman	77 (75.5)		39 (78.0)		38 (73.1)	
	Man	21 (20.6)		9 (18.0)		12 (23.1)	
	Nonbinary	4 (3.9)		2 (4.0)		2 (3.8)	
	Prefer not to say	0 (0)		0 (0)		0 (0)	
**Race, n (%)**							
	American Indian or Alaska Native	0 (0)		0 (0)		0 (0)	
	Asian	51 (50.0)		30 (60.0)		21 (40.4)	
	Black or African American	8 (7.8)		3 (6.0)		5 (9.6)	
	Multiracial	3 (2.9)		3 (6.0)		0 (0)	
	Native Hawaiian or Other Pacific Islander	0 (0)		0 (0)		0 (0)	
	Prefer not to say	7 (6.9)		1 (2.0)		6 (11.5)	
	White	33 (32.4)		13 (26.0)		20 (38.5)	
**Recent stress level, mean (SD)**			3.4 (0.9)		3.5 (0.8)		3.3 (1.0)
**Hours of sleep the night before the experiment, mean (SD)**			6.9 (1.1)		7.1 (1.0)		6.8 (1.1)
**Quality of sleep the night before the experiment, mean (SD)**			3.5 (0.8)		3.5 (0.8)		3.5 (0.8)
**Self-reported diagnosis of mental health condition, n (%)**							
	0 condition	66 (64.7)		32 (64.0)		34 (65.4)	
	1 condition	18 (17.6)		9 (18.0)		9 (17.3)	
	2 conditions	11 (10.8)		6 (12.0)		5 (9.6)	
	3 conditions	7 (6.9)		3 (6.0)		4 (7.7)	
**Individuals with diagnosed mental health conditions, n (%)**							
	Anxiety disorder	23 (22.5)		13 (26.0)		10 (19.2)	
	Attention-deficit/hyperactivity disorder	16 (15.7)		10 (20.0)		6 (11.5)	
	Bipolar disorder	1 (1.0)		0 (0)		1 (1.9)	
	Depression	11 (10.8)		4 (8.0)		7 (13.5)	
	Eating disorder	3 (2.9)		1 (2.0)		2 (3.8)	
	Obsessive-compulsive disorder	4 (3.9)		2 (4.0)		2 (3.8)	
	Psychosis	1 (1.0)		0 (0)		1 (1.9)	
	Posttraumatic stress disorder	1 (1.0)		0 (0)		1 (1.9)	
	Schizophrenia	1 (1.0)		1 (2.0)		0 (0)	

### Qualitative Results

#### Overview

**Figure 3:**
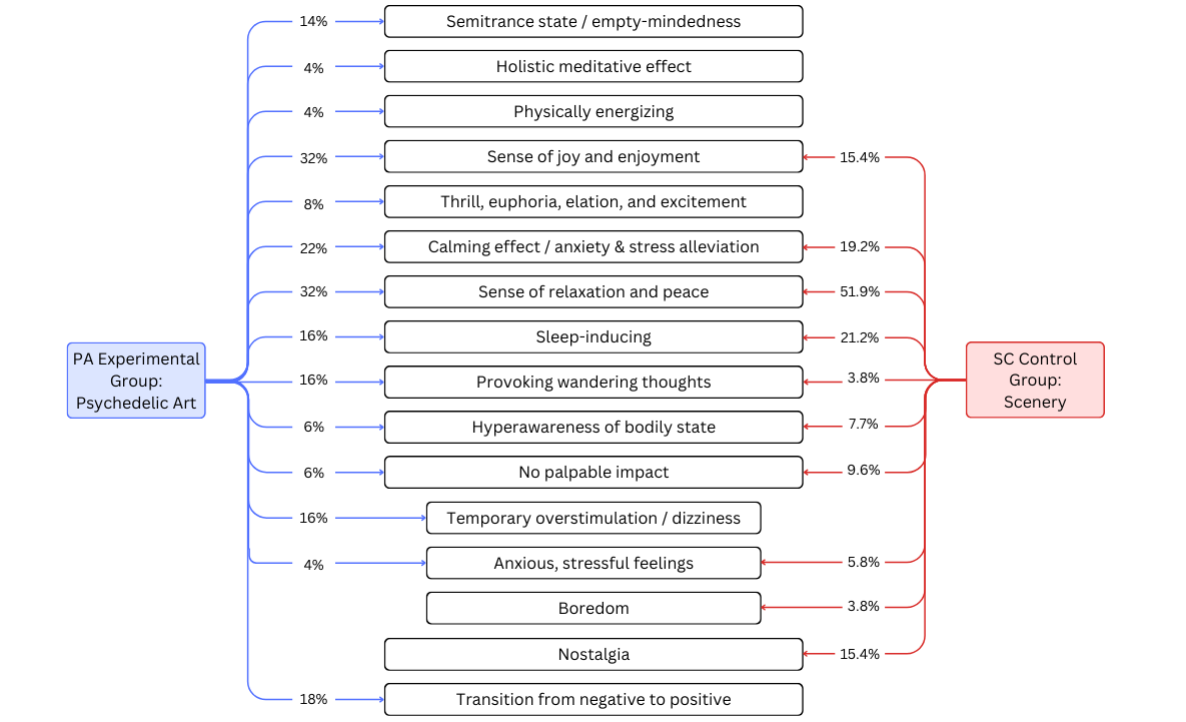
Comparative themes of the perceived effects between participants who viewed psychedelic art and participants who viewed natural scenery. The percentages indicate the proportion of each group that reported a specific theme.

The open-ended qualitative data on one’s physical, emotional, and mental states contributed substantial knowledge to the participants’ overall perception and experience during the experiment. Qualitative analysis unveiled a greater intensity and diversity of emotional, mental, and physical impacts induced by PA compared to natural scenery (Figure 3), including sense of relaxation and peace, anxiety and stress alleviation, joy, thrill and euphoria, sensations of awe and wonder, hypnotizing effect, holistic meditative effect, provocation of wandering and creative thoughts, induced hyperawareness of bodily states, and transitions from induced overstimulation or anxious thoughts to feelings of calmness. Illustrative quotations are provided in subsequent sections to support each theme (Table 2).

**Table 2:** Qualitative results on the perceived effects of viewing psychedelic art and natural scenery with illustrative quotation.

Themes	PA^a^ group	SC^b^ group
Relaxation and peace	“I physically felt very at ease.” (participant 76) “Gradually I was attracted to the dynamic effects of the video and [felt] a bit [of] emotional relaxed.” (participant 60)	“It was relaxing and gave space to take my mind off day to day activities.” (participant 99) “Relaxed. the 5 minutes went by very quickly, it felt like only a couple minutes passed.” (participant 86)
Enjoyment, entertainment, and joy	“There was so much cool stuff to see!!” (participant 5) “I felt entertained finding the patterns of the objects moving around.” (participant 9) “The bright colors made me feel happy and warm. The constant motion made me feel mesmerized.” (participant 30)	“The butterfly made me smile.” (participant 45) “It reminded me how beautiful the natural world is! It made me feel optimistic and happy to be a part of something as wonderful as Earth, and made me want to spend more time relaxing and reflecting on my own thoughts.” (participant 24)
Calming and decompressing effect	“I felt a lot calmer and less stressed while watching the video. I often struggle to calm my anxious thoughts, but I had a lot less negative and anxious thoughts while watching the video. Overall, I thought the video was very calming and stress-reducing, and I’d like to try using similar videos to de-stress in my own time.” (participant 42) “As a former cancer patient with mildly traumatic experiences with sedation that induced distressing psychedelic imagery, I was interested to see how this would impact my physical and emotional state. Soon into the video, persistent thoughts and stresses diminished significantly. I was extremely pleasantly surprised that the imagery throughout the video induced joy, relaxation, and peace.” (participant 11)	“I was a little worried and anxious when I started the experiment due to some personal issues I'm currently facing. I found images of nature and trees to be very soothing.” (participant 84) “I felt less tense watching the video and was able to forget about my stress for a while.” (participant 114)
Sleep-inducing effect	“Feeling hypnotized, a wave of calmness came over me, no intrusive anxious thoughts, my mind didn’t wander, my body felt like it was getting ready to sleep.” (participant 61)	“[It was] really peaceful and made me want to sleep.” (participant 97)
Wandering thoughts	“The video promoted thoughts about the universe, space, galaxies, plants like ferns and the family of vegetables that include cauliflower and broccoli. I also thought about cells, viruses, and other organelles and cell structures such as myosin and actin.” (participant 109)	“There were moments when I would let my mind wander.” (participant 43)
Semitrance states	“The physical state I felt I would describe as in a semi-trance like state.” (participant 109) “My mind became void of external thoughts.” (participant 76)	—^c^
Physical energization	“The bright colors made me feel warm and the constant motion made me feel energized.” (participant 22)	—
Hyperawareness of bodily states	“I was focused on my breathing, which I could regulate more.” (participant 103)	“Watching the scenes of nature while doing nothing gave me a chance to take deep breathes and just be.” (participant 59) “I became hyper aware of my breathing - and I found myself in a “zone” of sorts, where I realized I had been staring at one spot for a longer time than usual”. (participant 78)
Meditative effect	“The patterns that were more tunnel-like instead of swing-like were my favorite because they made me feel the most grounded and balanced” (participant 30).	—
Nostalgia	—	“I missed my family and hometown.” (participant 13) “Reminded me of home that I miss a lot.” (participant 84)
Neutral experiences	“I didn’t really feel a strong emotional response to it.” (participant 75)	“I don't find much change in my physical and emotional states.” (participant 51)
Negative experiences	“I felt less stressed for a second in the middle of the video where there was a flower-like pattern.” (participant 44) “I feel too much information captured by my eyes.” (participant 80) “A moment of dizzy when arts are in transition.” (participant 81)	“Hard to follow; some of the images were so stressful. Time lapse also more stressful.” (participant 4) “Some clips made me feel some vertigo when the video panned from up high.” (participant 52) “I felt like some clips bored me a little.” (SC participant 64)

^a^PA: psychedelic art.

^b^SC: scenery control.

^c^Not applicable.

#### Relaxation and Peace

A major theme reported by the PA experimental group participants was the sense of relaxation and peace while viewing PA (16/50, 32%). Participants commented on the “sense of being at ease,” “feeling mindful” of one’s “state of relaxation,” and “emotionally feeling relaxed since the environment was quiet and the visuals were appealing.” The same theme was also observed in the control group (27/52, 52%).

#### Enjoyment, Entertainment, Joy, and Excitement

Another common state described by the participants was the sense of entertainment, joy, and enjoyment induced by PA (16/50, 32%). In the PA group, participant commented on their sense of “awe,” “wonder,” and feelings of “happiness” and “ease” while viewing the “vibrant” and “vivid” colors and “fantastical patterns of PA. The similar motif of joy and enjoyment was also present in the control group (8/52, 15%). One participant in the control group described the scenery video as inducing “yearning for the outdoors, greatness, and adventure” (SC participant 88).

Among the PA participants that reported feelings of excitement, many described intense feelings of “thrill,” “euphoria,” and “audibly gasping” at the “mesmerizing visuals.” One participant recalled, “one of the sequences felt euphoric, like I was high” (PA participant 15). Another participant described the experience of viewing PA as “refreshing emotionally” (PA participant 17). In addition, one participant equated the experience of watching “dramatic changes in the patterns” as a less intense but “physically similar response to [being on] a roller coaster [that] was about to drop” (PA participant 111). No intense feelings of thrill or feelings of euphoria, elation, or exhilaration was reported in the control group.

#### Anxiety Alleviation and Decompressing Effect

A calming and decompressing effect that reduced existing anxiety, anxious thoughts, discomfort, or stress was reported by 22% (11/50) of the PA participants. The major difference between the reported calming effect and sensations of relaxation was that a calming effect was noted when participants explicitly described PA as mitigating stressful and anxious feelings, whereby a sense of relaxation and peace was noted when participants generally described their physical, emotional, or mental states as being at ease. One participant notes that the visuals allowed them to prevent “anxious thoughts [from] overtaking their mind and it felt much easier to distract their mind from anxious thoughts” by looking at PA (PA participant 110). One participant also described the association between the characteristics of PA visuals and the extent of the calming effect, noting that “when the colors and patterns took up the entire screen,” the calming effect was greater than “when the patterns were smaller and only took up part of the screen” (PA participant 42)*. Another reason for the calming effect reported by the participant was the ability of PA to absorb one’s attention, whereby the participant was “*focused so much on the visuals that they [I] did not hear the usual anxious thoughts as much” (PA participant 2). The same soothing impact was also observed in a similar proportion of the SC control group (10/52, 19%), whereby participants described how the video was able to alleviate their “stressful and anxious thoughts.”

#### Sleep-Inducing Effect

In addition, 16% (8/50) of the PA participants reported “hypotonization” and “sleep-inducing” as the primary impact of PA on their overall physical and mental states. A similar impact was observed in 21% (11/52) of the control participants.

#### Wandering Thoughts

Another impact on people’s mental states reported by several PA participants (8/50, 16%) was the ability of PA to induce “wandering thoughts.” Participants described how their minds started to “wander freely,” “wonder off,” and enter a free-flowing state. as they followed the moving patterns of PA. In addition, some participants also mentioned provoked thoughts about “the beautiful cosmos” (PA participant 29), “dreams last night” (PA participant 66), and “art and flowers” (PA participant 69). In comparison, induced wandering thoughts were only mentioned by 4% (2/52) of the participants in the control group, who commented on the scenery video “letting their mind wander” (SC participant 36).

#### Semitrance States

Meanwhile, some participants (7/50, 14%) reported a “semitrance” state induced by PA, commonly characterized by persistent feelings of empty-mindedness, complete absence of thoughts, a sense of total absorption and profound abstraction, or diminished or absent sensory activity. This theme was not observed in the SC control group.

#### Physical Energization

One palpable impact on physical states reported by the PA participants was the physically energizing quality of PA (2/50, 4%). The participants described their body feeling “energized” by the view of “the constant motion” of PA. One participant specifically noted that the video made their body feel energetic as they looked at the moving patterns and lines (PA participant 22). This theme was not reported by any participant in the control group.

#### Hyperawareness of Bodily States

Furthermore, 6% (3/50) of the PA participants emphasized that they became hyperaware of their bodily state as they viewed PA, such as heightened awareness of their heartbeat and breathing, intentional regulation of breathing, and mindful thinking about breathing. Similarly, 8% (4/52) of the participants in the control group commented on their increased mindfulness of their breathing, state of bodily tension, and heart rate.

#### Meditative Effect

A holistic and meditative impact entailing physical, emotional, and mental sensations was also reported by participants (2/50, 4%). A meditative state was noted when the participant described the impact of PA explicitly as meditative, inducing the intention or desire for meditation, or describing it as inducing a state of consciousness of sensation where the body and the mind were in harmony. This theme was not reported in the control group.

#### Nostalgia

One theme unique to the SC control group was the feeling of nostalgia induced by the scenery video, reported by 15% (8/52) of the participants. Images of “mountains, rivers, flowers, and tress” led participants to reminisce about memories of being outdoors and family or friends (SC participants 1, 46, and 79), homes and hometowns (SC participants 13, 43, and 84), memories of holidays (SC participant 49), and childhood (SC participant 56).

#### Neutral Experiences

Of the 50 PA participants, 3 (6%) reported neutral experiences, characterized by the lack of any strong or palpable changes in the participants’ physical, emotional, or mental states. Neutral responses, for example, included, “I wasn’t particularly agitated or excited” (PA participant 71) and “I didn’t really feel a strong emotional response to [psychedelic art]” (PA participant 75). A greater proportion of the control participants that viewed the scenery video reported neutral experiences (5/52, 10%). Nearly identical responses in the SC group, for example, included “there was not much of physical or emotional change” (SC participant 104) and “I don’t find much change in my physical and emotional states” (SC participant 51).

#### Negative Experiences

Negative sensations of overstimulation and sensory overload were reported by 20% (10/50) of the PA participants, whereby the “movements and colors” of PA induced mild feelings of stress, anxious thoughts, and dizziness. As one participant commented, “I felt that those bright and high saturation things tried to attract my attention and I felt stressful then staring at them” (PA participant 98). Another common theme among PA participants that reported negative sensations induced by PA was the transition from negative sensations, such as anxiety and stress, to positive sensations, such as calmness, relaxation, and mental engagement or a mixture of both positive and negative sensations (9/50, 18%). As one participant wrote, “I felt deep fear being aroused shortly into the video. It last for a few seconds. After that the feeling went away and I started to enjoy it” (PA participant 22). Mixed feelings of being transitorily “dizzy, relaxed, and refreshed” were also noted by one participant. Negative sensations of “vertigo” (1/52, 2%), “boredom” (2/52, 4%), and increased stress and anxiety (2/52, 4%) were reported by 5 control participants that viewed the scenery video.

## Discussion

### Principal Findings

This study provided preliminary evidence for the potential of PA to affect people’s emotional, mental, and physical states; qualitative data yielded substantial insights into participants’ embodied experiences of viewing PA. Induced changes in a wide variety of emotional, mental, and physical states were observed, including reduced anxiety and stress, sense of excitement, euphoria, joy, and thrill, hypotonization, enhanced feelings of relaxation and peace, increased energy, induced meditative thoughts, wandering thoughts, a semitrance state, hyperawareness of bodily states, and transitory feelings of overstimulation.

The brain responds to visual information in highly dynamic ways [33]. The combinations of various pathways of brain stimulation may contribute to the unique and often intense emotional responses associated with PA. This study showed that PA could evoke feelings of wonder. A few factors might contribute to this effect. PA contains abstract or surreal forms, which can challenge the viewer’s perception of reality, contributing to a sense of curiosity and wonder, as the viewer tries to make sense of what they are seeing. Finally, it should be noted that PA is often associated with the use of psychedelic drugs, which can induce altered states of consciousness and feelings of euphoria and wonder. The association with these experiences can create a sense of longing for these states, which can contribute to the positive emotional response to PA. The co-occurring emotional, mental, and physical impacts reported in the study may suggest increased functional connectivity between brain regions stimulated by the perception and processing of PA. If true, this increased connectivity may account for the sense of integration between different aspects of experience and the feeling of mind-body oneness and harmony reported in this study. In a study on motion sickness, researchers found significant increases in functional connectivity between the brain region that processes visual motion and the regions involved in nausea processing during experiences of motion sickness, revealing one of the many mechanisms in the brain that accounts for visual-somatic associations [34]. Concurrent physical-emotional impacts, such as inducing feelings of energization and semitrance state, that were uniquely imported in the PA experimental group raise questions on how the brain might process the visual information presented by PA differently than that of imagery of natural scenery.

Considering the potential ways in which the human brain responds to the compositions of PA, we may speculate several potential reasons for the unique and often intense emotional experiences associated with viewing this type of art. First, this art form often features bright and vibrant colors of high contrast, which can stimulate the visual cortex and evoke a sense of excitement and pleasure [35]. The use of bold colors can also create a sense of movement and energy, which could contribute to a feeling of euphoria. Second, research has shown that the perception of visual symmetry could spontaneously create positive affect and lead to affective congruence effects [36]. PA features intricate and repetitive patterns, which can create a sense of order and symmetry that consequently leads to feelings of pleasantness or more intense elation. It should be noted that the brain responds to PA in complex and varied ways, which can lead to a range of emotional and perceptual experiences. Visual stimulation, potentially increased connectivity between brain regions, and altered states of consciousness all offer promising areas for a deeper understanding of the unique and often intense emotional responses associated with PA.

Responses to open-ended questions regarding the embodied experience of viewing natural scenery showed less diverse and less intense impacts on people’s emotional, mental, and physical states. Compared to PA, no experiences of being physically energized, semitrance states, holistic meditative effects, intense sensations of euphoria, thrill, and elation were reported from viewing natural scenery. Previous research has speculated that the perception of abstract visual information reduced the predominant impact of familiar reality on the brain, allowing new emotional and cognitive connections to be created and allowing new associations or otherwise hard-to-access associations within the brain’s inner states to be activated, which could consequently induce diverse and rewarding sensations [37]. The greater diversity and intensity of psychosomatic responses induced by PA support this hypothesis.

### Implications for Mental Health

Evidence from this study suggests that PA could have a positive impact on mental health conditions by alleviating anxiety and stress and inducing relaxation, calmness, and sensations of joy and excitement. PA can thus be considered as a creative aid for psychotherapies and serve as a complementary therapeutic tool for the alleviation of psychiatric symptoms. The vibrant colors and intricate patterns can help to stimulate the imagination and enhance introspection, making it easier for individuals to access deeper emotions and thought patterns. Further evidence on whether viewing PA can facilitate emotional and psychological healing can also contribute to the development of novel art-based therapeutic strategies.

PA can be used to create immersive and therapeutic environments for individuals with mental health issues. For example, visualizations can be projected onto walls of people’s private rooms to promote relaxation and emotional well-being. PA can also be used to create interactive installations in hospitals and health care settings. By creating interactive installations that use colorful and imaginative visuals, hospitals can create a more engaging and immersive environment for patients and visitors. Hospitals and health care facilities can also offer immersive art therapy rooms that incorporate PA to promote mental health and well-being, where patients can engage in various art activities, including creating their own PA or exploring the art of others as a form of relaxation and self-expression.

As demonstrated by this study, besides a direct calming effect, PA can evoke a sense of transcendence, semitrance, or a feeling of being connected to something larger than oneself. This sense of transcendence can promote a sense of well-being and reduce stress levels. Given the induced meditative state reported in this study, PA can also be used as part of mindfulness and meditation practices to promote relaxation and focus. The intricate patterns and bright colors can help to calm the mind and enhance focus, making it easier to achieve a meditative state for stress reduction. The direct and indirect associations between PA and stress suggest that viewing or creating PA can be a tool for reducing anxiety and stress levels and promoting relaxation and well-being. However, given the few reported cases of overstimulation and passing feelings of anxiety and stress, more research is needed to fully understand how PA can be tailored to reduce anxiety and stress levels.

While there is limited research on the specific use of PA for the treatment of sleep disorders, the sleep-inducing quality of PA reported in this study illuminates a few potential ways that it could be used to improve sleep or treat sleep disorders. First, PA can be used for relaxation and mindfulness exercises. Incorporating PA into a bedtime routine, such as viewing it before going to sleep, could help to reduce stress and anxiety and promote better sleep. The association between PA and dreams reported in this study also suggests that PA could contain imagery that is reminiscent of dreams or altered states of consciousness. Incorporating this form of art into a bedtime routine may help simulate a dream-like state that induces sleep. Third, PA can be visually stimulating for many and evoke positive emotions, which may help to improve mood and reduce negative thoughts or feelings that can interfere with sleep. Particularly for people with sleep disorders such as insomnia, it can be helpful to have a distraction from negative thoughts and worries that can interfere with falling asleep. PA may be a visually engaging and distracting tool that can help to redirect attention away from negative thoughts and promote relaxation.

Previous research has shown that music has a significant therapeutic effect in psychedelic therapy [38], a form of therapy where psychedelic drugs are used to induce emotional release [39]. Given the significant impact on people’s emotional and mental states reported in this study, future research could investigate whether PA plays a similar therapeutic function in psychedelic therapy and whether it can help patients explore and process their psychedelic experiences in a therapeutic setting.

### Implications for Digital Mental Health Tools

Research has shown increased use of digital technologies for art therapy over the years [40]. In a recent study on improving well-being through interaction with digital art, Trupp and colleagues [41] showed that participants who engaged with web-based art exhibitions even with just 1 to 2 minutes of exposure reported better mood and a reduced sense of anxiety and loneliness. Given its positive emotional and mental impacts reported in the study, PA can also be considered for integration into mobile mental health applications as an innovative and *cost-effective means to promote mental health* [42]. First, it can be used for mood enhancement by providing users with colorful and visually stimulating images that evoke positive emotions. This could potentially help to reduce symptoms of anxiety and depression. Second, as suggested by Golden and colleagues [43], settings and esthetics played an important role in improving psychedelic therapies and their delivery. Therefore, PA can be considered for integration into applications designed to support patients undergoing psychedelic therapy. PA can be used as a visual aid to help users explore their thoughts and emotions and facilitate a deeper understanding of their psychedelic experiences. Third, as revealed by a recent study on evidence-based commercial mental health mobile apps, app esthetics (ie, user interface design) showed significant association with consumer appeal [44]. Given its general esthetic quality, PA could be used to enhance the overall esthetic appeal of the app, making it more engaging and enjoyable to use. This could help to increase user retention and engagement. Finally, with a rich cultural history, PA is deeply rooted in the counterculture movement of the 1960s [3]. By incorporating it into mobile mental health apps, developers could incorporate cultural elements and promote a sense of community among users.

Digital PA, if incorporated into mobile health apps, can be readily viewed on a mobile phone, tablet, or computer. However, it should also be noted that individuals without access to electronic devices with access to sufficient mobile data or Wi-Fi, may face low accessibility in the use of features that require faster internet speeds. Digital divide and digital health accessibility are intricately connected [45]. Individuals with limited access to smart devices due to disabilities, cost of devices, limited digital literacy, and insufficient internet connection will likely face the same disadvantage in using PA as a digital health tool. Therefore, similar to other interventions in the digital health space, governments, health workers, and designers must deliberate on how to ensure accessibility to the intended users of designed art–based digital health tools.

This study has also shown that viewing PA can induce a wide variety of emotional, mental, and physical impacts. A “one-size-fits-all” PA-based therapeutic tool or intervention will likely not be effective for all, which prompts deliberation on how, if it is to be used for health and therapeutic purposes, we can design user-centered interventions that tailor and maximize the positive impact of PA for individuals. Integrating artificial intelligence (AI), which has been shown as an effective means to aid personalized health management and optimize health outcomes [46], can similarly be considered for PA-based digital health tools to develop user-centered therapeutic strategies. Specifically, AI algorithms can be used to generate novel and complex patterns and color combinations that are characteristic of PA to avoid repetitions, maximize engagement, and thereby potentially maximizing the therapeutic effect of PA viewing. AI algorithms can be further incorporated into PA-aided therapy sessions by generating art that is tailored to the individual’s preferences and needs. For example, an AI system could generate art that is specifically designed to induce feelings of calmness or happiness based on the user’s responses to certain stimuli. If data can be collected on what baseline colors, motions, and patterns are perceived to be the most esthetically pleasing, engaging, and inducing the most positive experience for different individuals, AI algorithms can be designed to generate PA using these inputs. If PA is to be incorporated into psychotherapeutic settings, a neurofeedback algorithm can be designed where patients’ real-time electrocardiogram data can be fed into an AI-algorithm that generates PA to induce maximum experiences of relaxation, calmness, excitement, or meditative states. The implementation of this potential strategy necessitates more research on the neural activities associated with people’s emotional states. In addition, AI can also be used to increase accessibility. AI-generated PA can be made available on the web, allowing people who are unable to access traditional art therapy services to enjoy the mental health benefits of PA. This can be particularly beneficial for people living in rural areas or those who have mobility or financial constraints.

### Future Research

Medical research on PA is a relatively new area of study. Given the insights from this study, there are several important directions that researchers could pursue to further advance this field. First, research can explore the efficacy of PA therapy in treating specific mental health conditions. This includes investigating the therapeutic mechanisms of PA therapy, identifying patient populations that may benefit the most from this approach, and developing standardized protocols for delivering PA therapy.

Second, research can also explore the neural correlates of PA, including the brain regions and neural networks that are activated during the viewing of PA, as well as the potential for PA to induce altered states of consciousness. For example, a previous study has shown that addiction and substance abuse were primarily due to the induced biological rewards through the substance’s ability to stimulate endogenous brain circuitry [47]. By determining the brain circuitry activated by PA and exploring the potential of engineering it to interfere with the habitual endogenous brain circuitry of addiction patients undergoing rehabilitation, PA could potentially be used as a creative noninvasive harm reduction tool.

In addition, the integration of PA into health care settings could also benefit a deeper understanding through implementation science research. Research can explore best practices for integrating PA into health care settings, including hospitals and clinics. This includes identifying the most effective methods for displaying PA, developing guidelines for creating healing environments, and studying the effects of PA on patient outcomes.

### Limitations

This single-blind exploratory study aimed to minimize researcher bias by having the analyst unaware of group assignments, although participants could not be blinded due to the distinguishable nature of the PA and natural scenery videos they viewed. To reduce bias, participants received no information about expected impacts. The study’s generalizability was limited by age restrictions and an unequal distribution of racial and gender identities, with certain groups underrepresented. Future research could explore if significant variation exists across the impact of PA on different populations, including older adults and varied socioeconomic backgrounds. While the goal of the study was to provide personal insights into the emotional, mental, and psychological impacts of PA, which are difficult to quantify through objective measures alone, we acknowledge that while the qualitative approach allowed us to obtain rich subjective experiences, future studies could benefit from adopting a mixed methods approach that integrates both qualitative and quantitative data. Including validated scales and neurobiological indicators, alongside in-depth self-reported descriptions, such as those measuring anxiety or emotional arousal, could provide additional layers of reliability and facilitate larger-scale research to build upon the in-depth insights provided by this study. In addition, the impact of PA on creativity, cognitive abilities, and specific mental health conditions warrants further investigation. Nonetheless the study’s findings suggest that PA could be used as a tool for creative expression and therapeutic purposes, particularly in meditative and spiritual settings, offering new avenues for enhancing mental and emotional well-being. In addition, as this was an exploratory study, the imageries were sourced based on available artistic resources and general principles associated with PA and natural scenery. The content decisions were made with the goal of maximizing contrast between the experimental and control groups but were constrained by practical limitations, such as the availability of preexisting PA content. However, this provides a venue for future research to explore how studies at the intersection of arts and health could engage in interdisciplinary collaboration between designers and health researchers to develop nuanced and tailored creative content that align closely with both artistic and scientific objectives, potentially enhancing the depth of future findings. Finally, this study examined the impact of PA in a laboratory setting, where participants did not partake in any other activity while viewing PA. Given the transpersonal experiences, such as feelings of interconnectedness, mind-body harmony, and semitrance states, documented by this study, future research could explore the psychological effects of PA in more diverse settings, such as meditative and spiritual activities and examine if PA could enhance the experience of such activities.

### Conclusions

This study provided preliminary evidence for a wide variety of emotional, mental, and physical impacts perceived by individuals upon viewing digitally rendered PA, including induced sensations of relaxation and peace; anxiety and stress alleviation; sensations of joy, excitement, and euphoria; sensations of awe and wonder; hypnotizing effect; holistic meditative effect; provocation of wandering and creative thoughts; induced hyperawareness of bodily states; and transitions from induced overstimulation or anxious thoughts to feelings of calmness. Responses on participants’ embodied experiences and perceptions show greater diversity and intensity of people’s psychosomatic responses. Overall, the findings suggest that PA is a rich and complex form of visual art that has the potential to inspire creativity, facilitate healing, and promote well-being.

## Appendix

Multimedia Appendix 1 All video files generated for this study.

Multimedia Appendix 2 CONSORT (Consolidated Standards of Reporting Trials) checklist.

## Abbreviations

AI: artificial intelligence
LSD: lysergic acid diethylamide
PA: psychedelic art
SC: scenery control
