# Supplementary material for: Psychedelic Art and Implications for Mental Health: Randomized Pilot Study
Source: JMIR Form Res. 2024 Dec 3;8:e66430. doi: 10.2196/66430 (PMC11653042; doi:10.2196/66430)
Supplement: Multimedia Appendix 1 [file formative_v8i1e66430_app1.docx]

# Video Materials:

**Link to psychedelic art used in the experimental group:**

<https://drive.google.com/file/d/16ODkSNUnVQ0BRzaAo321T8IFkY5ID5ey/view>

(Created with clips from publicly available and free psychedelic art footages. Source: Psychedelic Videos. “4K Psychedelic Animated Graphics - 2 Hours!”.YouTube. Jul 24, 2019. https://www.youtube.com/watch?v=bx-0YlFprqc)

**Link to natural scenery used in the control group:**

<https://drive.google.com/file/d/1EP792vEJ7GJS93Dzx4N8cHFxb7CZ0C3E/view>

(Created from natural scenery stock videos using Canva Pro)
